# Vibrational characteristics of iVAC and three ultrasonic metallic inserts: analysis by laser Doppler vibrometry

**DOI:** 10.1590/1807-3107bor-2025.vol39.040

**Published:** 2025-04-04

**Authors:** Hermano Camelo PAIVA, Eduardo AKISUE, Marco Antônio Húngaro DUARTE, George Táccio de Miranda CANDEIRO, Marcílio ALVES, Giulio GAVINI

**Affiliations:** (a) Universidade de São Paulo – USP, School of Dentistry,Department of Restorative Dentistry, São Paulo, SP, Brazil.; (b) Universidade Santa Cecilia – Unisanta, School of Dentristry, Discipline of Endodontics, Santos, SP, Brazil.; (c) Universidade de São Paulo – USP, School of Dentristy, Department of Dentistry, Endodontics, and Dental Materials, Bauru, SP, Brazil.; (d) Centro Universitário Christus – Unichristus, Department of Restorative Dentistry, Fortaleza, CE, Brazil.; (e) Univesidade de São Paulo – USP, Department of Mechatronics and Mechanical Systems Engineering, São Paulo, SP, Brazil.

**Keywords:** Endodontics, Root Canal Irrigants, Root Canal Therapy

## Abstract

The iVAC system combines ultrasonic activation with negative-pressure irrigation, highlighting the importance of determining its vibration characteristics. This study aimed to evaluate the oscillation characteristics of the iVAC system using laser Doppler vibrometry and to compare them with those of metallic inserts used for ultrasonic irrigation. Four ultrasonic inserts—Irrisonic, Nitisonic, Ultra X Blue, and iVAC—were attached to an ultrasonic unit, secured in a holder, and operated at a power setting of 15%. A laser scanning vibrometer was used to scan the first four millimeters of each insert. Each measurement, lasting approximately 20 seconds, was repeated ten times per insert and performed at millimeter intervals. Displacement amplitude and frequency data were recorded for each insert. Furthermore, scanning electron microscopy was employed to evaluate the surface finish of the inserts, and their elemental compositions were analyzed using energy-dispersive X-ray spectroscopy (EDS). Analysis of variance and Tukey’s test were conducted to compare the performance of the inserts. The displacement amplitudes showed significant differences between inserts and among the scanned points (p < 0.05). All inserts demonstrated ultrasonic vibration frequencies exceeding 20 kHz. EDS analysis revealed that the elemental composition of the E1-Irrisonic tips was consistent with stainless steel. In contrast, the Nitisonic insert displayed a chemical composition characteristic of an equiatomic nickel–titanium (NiTi) alloy, whereas the Ultra X Blue insert contained additional elements beyond NiTi. The characteristics and composition of ultrasonic inserts significantly influence their displacement amplitude and vibration frequency. At the tested activation power, the iVAC insert, made of the organic thermoplastic polymer polyether ether ketone (PEEK), demonstrated an ultrasonic vibration pattern but exhibited the lowest oscillation amplitude among the inserts.

## Introduction

Effective cleaning and disinfection of the root canal system are crucial for the success of endodontic therapy.^1,2^ The efficiency of the irrigation system relies on the ability of the solution to reach non-instrumented regions and hard-to-access areas while generating sufficient flow and reflux to transport dentin debris toward the cervical region of the root canal.^3,4^

Although manual irrigation with positive pressure is the most commonly used method, it has certain limitations. One such limitation is the formation of bubbles in the apical third of root canals, which prevents the irrigating solution from reaching this region—a phenomenon referred to as “vapor-lock.”^5,6^ Another limitation of this irrigation method is the creation of a “dead water zone,”^3^ where the irrigant reaches the apical third but is not effectively renewed.

The limitations of manual positive pressure irrigation can be addressed using agitation techniques that enhance the penetration of irrigating solutions, leading to improved cleaning^7^ and disinfection of the root canal.^8^ Ultrasonic activation is the most frequently employed method for increasing the mechanical and chemical efficacy of irrigants.^4^ Another approach is negative pressure irrigation, in which the irrigant is delivered into the pulp chamber and aspirated through a suction cannula within the root canal, reaching the working length. This technique maintains a consistent irrigation flow from the pulp chamber to the apical region.^9^

The iVAC^®^ system is an irrigation device that integrates negative pressure irrigation with ultrasonic vibration. It features a microcannula made primarily of PEEK, a colorless organic thermoplastic polymer. PEEK is widely used as a biomaterial for manufacturing spinal, orthopedic, and trauma implants owing to its inertness, rigidity, durability, and resistance to chemical and mechanical stresses.^10^ The iVac microcannula is attached to an ultrasonic handpiece via a metal insert and connected to tubing that facilitates suction through a vacuum mechanism. The irrigant is introduced into the pulp chamber either directly from the ultrasonic device reservoir or using an irrigation cannula and syringe.

The cross-section, length, taper, and composition of ultrasonic inserts are known to affect their oscillation characteristics.^11,12^ In turn, these oscillation characteristics influence the flow of irrigant around the insert. Understanding the oscillation pattern, amplitude, and frequency of vibration is therefore essential for analyzing the behavior of the insert and the resulting flow, which are key factors in the cleaning and disinfection of the root canal system using both current and newly designed devices.

No studies have yet evaluated the vibrational patterns of inserts made from PEEK, highlighting the need to understand the behavior of this material. This study aimed to assess the oscillation characteristics of the iVAC system using laser Doppler vibrometry and to compare them with those of inserts commonly used for this purpose. The null hypothesis proposed that the tip type does not affect the amplitude or frequency of oscillation and that all inserts exhibit the same oscillation pattern.

## Methods

### Laser Scanning Vibrometry Analysis

**Figure 1:**
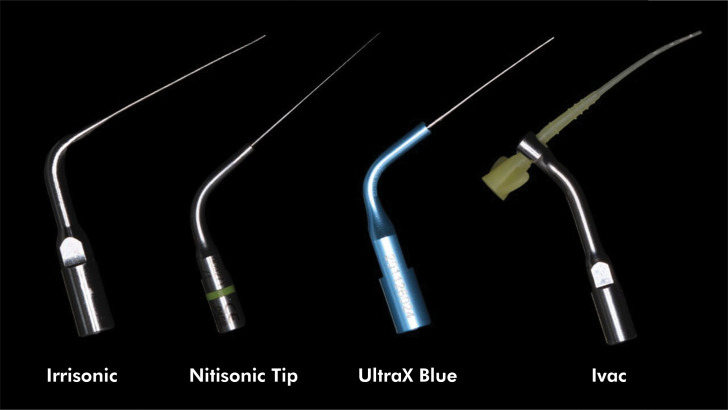
Inserts used in the study.

Four ultrasonic inserts were evaluated in this study: Irrisonic (Helse, Santa Rosa de Viterbo, São Paulo, Brazil), Nitisonic (Ultradent, South Jordan, , USA), Ultra X Blue (Eigtheeth, Changzhou, Jiangsu, China), and iVAC (Pac-Dent, Brea, , USA) (Figure 1). The displacement amplitudes along the first four millimeters of their lengths were measured using a laser scanning vibrometer (model OFV-323 High Frequency Scanning Vibrometer System, Polytec GmbH, Polytec-Platz, Waldbronn, Germany) and a vibrometer controller (model OFV-3020 – Vibrometer Controller System, Polytec GmbH, Polytec-Platz, Waldbronn, Germany). A laser Doppler vibrometer (LDV) measures the Doppler frequency shift of a laser beam scattered from an oscillating object to determine its vibration speed.^13,14^ MATLAB software (MathWorks Inc., New Mexico, USA) was used to calculate the vibration displacement amplitude and frequency from the velocity data.

**Figure 2:**
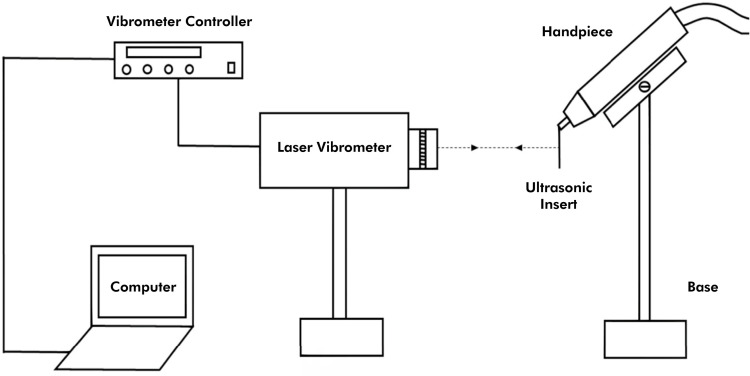
Representative illustration of the experiment.

**Figure 3:**
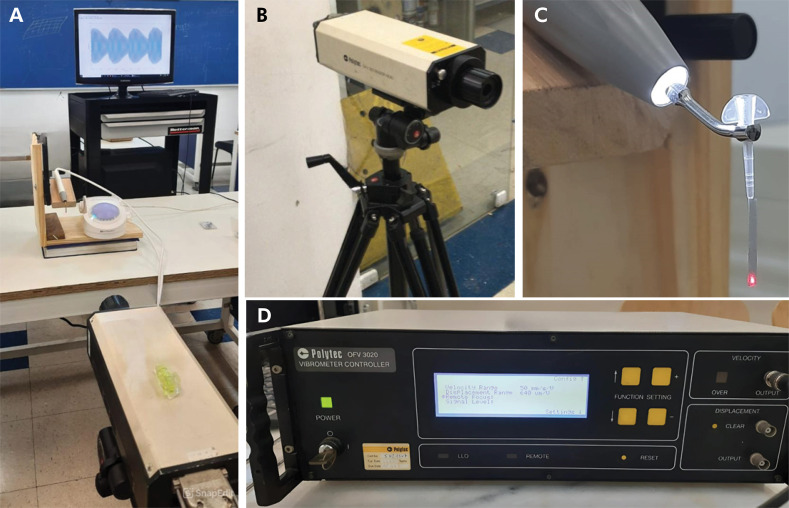
A.: Execution of the experiment, using the laser Doppler vibrometer. B.: Laser model OFV-323. C.: Handpiece positioned on the support for data acquisition. D.: Vibrometer controller model OFV-3020.

All ultrasonic inserts were operated using an Ultrawave XS ultrasonic scaler (Ultradent, South Jordan, USA). The handpiece was secured to a stand to ensure that the long axes of the inserts remained vertical and perpendicular to the laser beam (Figures 2 and 3). The stand, equipped with a millimeter ruler, allowed the laser to target four specific points along the inserts, spaced 1 mm apart.

The Ultrawave XS ultrasonic scaler allows power settings to be adjusted between 1 (minimum power) and 20 (maximum power). For this study, three repeated scans were conducted on the inserts at level 3 (15% power) within a frequency measurement range of 0 to 125 kHz. Within this range, the LDV operated with a sampling frequency of 250 kHz and a frequency resolution of 20 Hz.

The LDV was configured to perform ten repeated readings at each scanning point, with each scan lasting approximately 20 seconds and separated by 10-second intervals. Displacement velocity data from all four scan points on each insert were recorded and used to calculate the average displacement amplitude at each point. The entire procedure was conducted in duplicate for each insert studied.

Statistical analysis was performed using Jamovi software (Version 1.6.23.0). Data normality was evaluated using the Shapiro-Wilk test. As the data followed a normal distribution, displacement amplitudes at each scan point were analyzed using one-way analysis of variance with Tukey’s test for multiple comparisons. A significance level of p < 0.05 was applied.

### Metallurgical Analysis

Before use, a preliminary visual inspection of the metal inserts was conducted to check for any conspicuous defects. The design of the insert tips was then examined using scanning electron microscopy (LEO 440, Leica-Zeiss, Cambridge, UK) at magnifications of 100× and 350×. The chemical composition of each ultrasonic insert was analyzed semi-quantitatively using energy-dispersive X-ray spectroscopy (Oxford X-Max 80 EDX, Oxford Inc., Oxfordshire, UK). Three inserts from each manufacturer were included in both tests.

The specimens were systematically positioned and securely anchored into scanning electron microscope stubs. The parameters for the energy-dispersive X-ray spectroscopy analysis were as follows: an accelerating voltage of 20 kV, a beam current of 110 μA, a pressure of 10^-6^ Torr, an analysis area of 130 × 130 μm, an acquisition time of 200 seconds, and a detector dead time ranging from 30% to 35%. For each insert type, three samples were analyzed, with two areas selected from each sample, resulting in a total of six analyses per group.

## Results

### Laser Scanning Vibrometry Analysis

The results indicated significant differences in oscillation among the inserts and across the scanned millimeters (p < 0.05). In general, the highest oscillation amplitudes were observed in the first millimeter of each insert, with amplitudes decreasing progressively in the second, third, and fourth millimeters (Table). However, the Irrisonic and Ultra X Blue inserts exhibited their maximum oscillation amplitudes in the third millimeter, measuring 7.51 µm and 5.60 µm, respectively.

**Table:** Displacement amplitude data (mean ± standard deviation) along the 4 mm length of each ultrasonic insert.

Displacement (µm)				
Insert	Scanned point			
	1 mm	2 mm	3 mm	4 mm
Irrisonic	3,38 (± 0,089)^Aa^	2,82 (± 0,154)^Ab^	7,51 (± 0,209)^Ac^	3,81 (± 0,150)^Ad^
Nitisonic	5,50 (± 0,105)^Ba^	3,95 (± 0,054)^Bb^	3,89 (± 0,0502)^Bc^	4,9 (± 0,0272)^Bd^
UltraX Blue	4,83 (± 0,171)^Ca^	1,63 (± 0,220)^Cb^	5,60 (± 0,290)^Cc^	4,59 (± 0,112)^Cd^
iVAC	2,91 (± 0,0807)^Da^	1,18 (± 0,129)^Db^	1,01 (± 0,104)^Dc^	0,976 (± 0,047)^Dd^

Different capital letters indicate differences between the groups studied. Different lowercase letters indicate differences between the points scanned in the same group.

**Figure 4:**
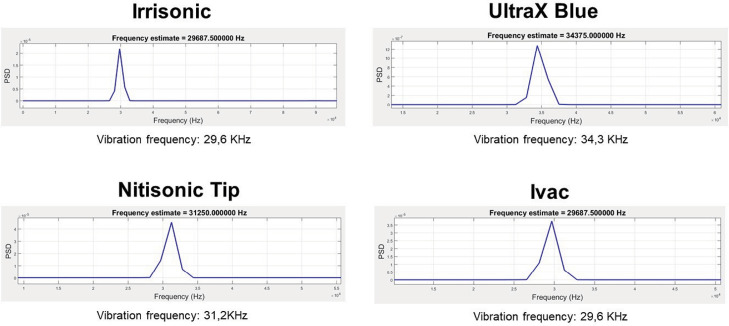
Frequency spectrum obtained after scanning the inserts at power setting 3 (15%). Large peaks are observed at frequencies above 20 kHz on all inserts.

All inserts demonstrated vibration frequencies above 20 kHz, qualifying them as ultrasonic. The frequencies ranged from 29.2 kHz to 34.1 kHz (Figure 4).

The Irrisonic insert exhibited oscillation amplitudes ranging from 2.82 µm to 7.51 µm and a vibration frequency of 29.6 kHz. The Nitisonic insert showed oscillation amplitudes between 3.89 µm and 5.50 µm, with a frequency of 31.2 kHz. The Ultra X Blue insert had oscillation amplitudes ranging from 1.63 µm to 5.60 µm and a frequency of 34.3 kHz. The iVAC insert displayed oscillation amplitudes ranging from 0.97 µm to 2.91 µm, with a vibration frequency of 29.6 kHz, matching that of the Irrisonic insert.

### Metallurgical Analysis

**Figure 5:**
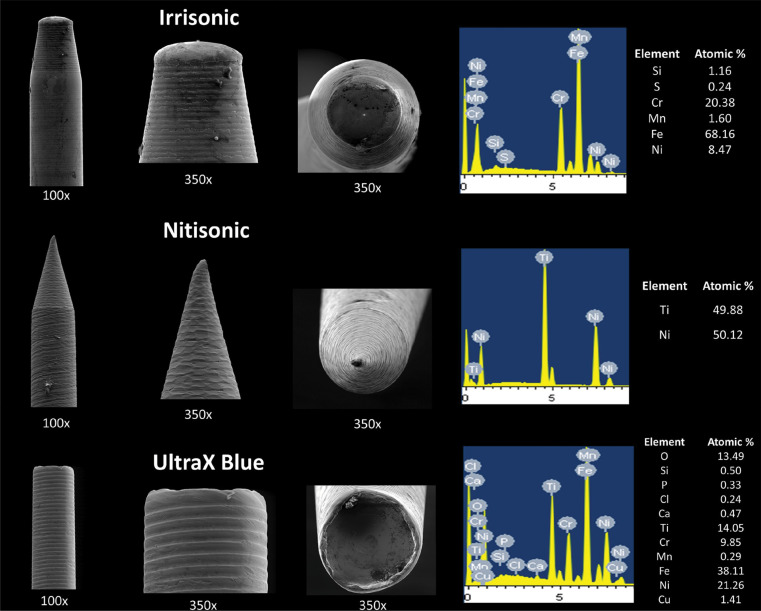
Scanning Electron Microscopy and Energy Dispersive Spectroscopy of the Irrisonic, Nitisonic and UltraX Blue inserts.

Scanning electron microscopy revealed that the Irrisonic and Ultra X Blue inserts have flat tips, whereas the Nitisonic insert features a pyramidal tip without sharp edges. Energy-dispersive X-ray spectroscopy analysis of the Irrisonic insert showed that it was composed of iron (68.16%), chromium (20.38%), and nickel (8.47%), consistent with typical surgical steel alloys. The absence of detectable carbon suggests lower susceptibility to corrosion. Traces of manganese, silicon, and tin were also identified (Figure 5). The Nitisonic insert was primarily composed of nickel (50.12%) and titanium (49.88%), indicating a nickel–titanium (NiTi) alloy (Figure 5). The Ultra X Blue insert consisted of iron (38.11%), nickel (21.26%), titanium (14.05%), oxygen (13.49%), chromium (9.85%), and copper (1.41%). Smaller amounts of silicon, chlorine, calcium, phosphorus, and manganese were also detected (Figure 5).

## Discussion

Understanding the oscillation patterns of ultrasonic inserts is essential, as these characteristics^11,15,16^ determine the biophysical interactions involved in ultrasonic irrigation. Factors such as the cross-section, length, taper, and material composition of the insert significantly influence oscillation dynamics.^11,12^

Within the limitations of this study, the null hypothesis was rejected, as the results demonstrated that the characteristics and composition of the ultrasonic inserts affected both the displacement amplitude and vibration frequency.

The laser scanning vibrometry technique used in this investigation did not include concurrent irrigation.^12^ The inserts were allowed to oscillate freely in open air because the laser focal point of the vibrometer was too wide, which would have compromised the effectiveness of signal capture. However, this method can be adapted to better simulate clinical conditions by incorporating simulated canals, as demonstrated in previous studies.^17^

Biophysical forces, such as transient and stable cavitation (acoustic streaming), rely on displacement amplitude. These forces typically occur when the ultrasonic insert vibrates freely with displacement amplitudes exceeding 135 µm.^18,19^ However, our results showed maximum displacement amplitudes of up to 7.5 µm, likely owing to the low power setting (15%) used in the evaluation. Increasing the power was not feasible because the absence of simultaneous irrigation would have caused overheating at higher power levels, potentially melting the iVAC polymer cannula. The highest power setting that avoided melting the cannula was selected to enable comparison across the inserts. Further studies that simulate clinical conditions, incorporating irrigation solutions and confined spaces, are needed to gain a more comprehensive understanding of the performance of these inserts.

All the analyzed inserts demonstrated frequencies exceeding 20 kHz, classifying them under ultrasonic frequencies. The Ultra X Blue and Nitisonic inserts exhibited the highest vibration frequencies, at 34.3 kHz and 31.2 kHz, respectively. In contrast, the Irrisonic and iVAC inserts were at the lower end of the frequency spectrum, both measured at 29.6 kHz.

The NiTi alloy used in two of the studied inserts can undergo various heat treatments, which alter its crystalline structure and result in different mechanical properties. Standard NiTi alloys, without heat treatment, exhibit superflexibility, enabling the instrument to return to its original shape after the load is removed. Conversely, instruments manufactured using heat-treated NiTi alloys may exhibit the shape-memory effect, which allows the alloy to return to its pre-deformation shape when heated above the austenite-to-martensite transformation temperature.^20^

The Nitisonic insert is made from a conventional NiTi alloy without heat treatment, making superflexibility its primary characteristic. In contrast, the Ultra X Blue insert contains a heat-treated NiTi alloy, which imparts a shape-memory effect. The Irrisonic insert is made from a surgical steel alloy, a material commonly used in most ultrasonic inserts. The primary motivation for this study was the unique composition of the iVAC insert, which, unlike traditional ultrasonic inserts used for canal activation, is made from the polymeric material PEEK.

In general, inserts made from metal alloys exhibited higher oscillation amplitudes. This can be attributed to the mechanical properties of these materials, as the modulus of elasticity of steel and NiTi alloys is significantly greater than that of polymeric materials.^11^ However, other factors, such as the cross-section, taper, and length of the insert, can also influence the oscillation pattern.^11,12,21^

Scanning electron microscopy revealed differences in the geometry of the insert tips. The Irrisonic and Ultra X Blue inserts featured flat tips, whereas the Nitisonic insert had a pyramidal tip without sharp edges. Although these inserts are designed for use in already-enlarged root canals, the presence of flat edges may contribute to uncontrolled dentin wear,^22,23^ a potentially negative outcome that warrants further investigation in future studies.

Energy-dispersive X-ray spectroscopy analysis revealed that the Irrisonic and Nitisonic inserts had chemical compositions consistent with stainless steel and NiTi alloys, respectively. As expected, the Ultra X Blue insert contained both nickel and titanium. The presence of oxygen in a significant percentage (13.49%) likely results from a titanium oxide layer formed during the heat treatment of this insert.^18^ Notably, iron (38.11%) was the most abundant element, with smaller amounts of chromium (9.85%), suggesting that this insert is composed of a combination of NiTi alloys and stainless steel.

The modulus of elasticity of nitinol ranges from 28–41 GPa in the martensitic phase and 52–75 GPa in the austenitic phase.^24^ Combining nitinol with stainless steel, which has a higher modulus of elasticity,^25^ would increase the overall stiffness of the insert. Therefore, the Ultra X Blue insert would be expected to exhibit a higher natural frequency than an insert made solely from nitinol, but potentially similar to or slightly lower than one made entirely from stainless steel. However, the results indicated that the Ultra X Blue insert had a higher vibrational frequency compared with the Irrisonic insert. This difference may result from a synergistic interaction between the two metal alloys or from the more uniform and effective distribution of stresses facilitated by the properties of NiTi. To gain a deeper understanding, further studies involving microstructural tests, phase analyses, and mechanical evaluations are needed.

Ultrasonic activation, negative-pressure irrigation, and continuous irrigation are strategies designed to address the limitations of manual positive-pressure irrigation.^4,7,26^ The iVAC system is particularly promising as it integrates all three strategies into a single device. The results of this study do not provide sufficient evidence to determine which insert is most effective at generating an acoustic stream and transient cavitation to enhance cleaning and disinfection efficiency. However, previous research has demonstrated that higher oscillation amplitudes are associated with improved canal cleaning.^27^

Further studies are needed to evaluate the effectiveness of the iVAC system in cleaning and disinfecting root canal systems.

## Conclusion

At the activation power used, the iVAC exhibited an ultrasonic vibration pattern, despite being predominantly composed of the organic thermoplastic polymer PEEK. However, it displayed the lowest oscillation amplitude compared with the metallic inserts. The frequency and variation in power observed in the metal tips were influenced by the composition of their respective metal alloys.
